# Rational engineering of *Geobacter sulfurreducens* electron transfer components: a foundation for building improved *Geobacter*-based bioelectrochemical technologies

**DOI:** 10.3389/fmicb.2015.00752

**Published:** 2015-07-30

**Authors:** Joana M. Dantas, Leonor Morgado, Muktak Aklujkar, Marta Bruix, Yuri Y. Londer, Marianne Schiffer, P. Raj Pokkuluri, Carlos A. Salgueiro

**Affiliations:** ^1^Research Unit on Applied Molecular Biosciences (UCIBIO), Rede de Química e Tecnologia, Departamento de Química, Faculdade de Ciências e Tecnologia, Universidade Nova de LisboaCaparica, Portugal; ^2^Department of Biological Sciences, Towson UniversityTowson, MD, USA; ^3^Departamento de Química Física Biológica, Instituto de Química-Física “Rocasolano”, Consejo Superior de Investigaciones CientíficasMadrid, Spain; ^4^Biosciences Division, Argonne National LaboratoryLemont, IL, USA

**Keywords:** *Geobacter*, cytochrome *c*, multiheme, extracellular electron transfer, *Geobacter* mutant strains

## Abstract

Multiheme cytochromes have been implicated in *Geobacter sulfurreducens* extracellular electron transfer (EET). These proteins are potential targets to improve EET and enhance bioremediation and electrical current production by *G. sulfurreducens*. However, the functional characterization of multiheme cytochromes is particularly complex due to the co-existence of several microstates in solution, connecting the fully reduced and fully oxidized states. Over the last decade, new strategies have been developed to characterize multiheme redox proteins functionally and structurally. These strategies were used to reveal the functional mechanism of *G. sulfurreducens* multiheme cytochromes and also to identify key residues in these proteins for EET. In previous studies, we set the foundations for enhancement of the EET abilities of *G. sulfurreducens* by characterizing a family of five triheme cytochromes (PpcA-E). These periplasmic cytochromes are implicated in electron transfer between the oxidative reactions of metabolism in the cytoplasm and the reduction of extracellular terminal electron acceptors at the cell's outer surface. The results obtained suggested that PpcA can couple e^−^/H^+^ transfer, a property that might contribute to the proton electrochemical gradient across the cytoplasmic membrane for metabolic energy production. The structural and functional properties of PpcA were characterized in detail and used for rational design of a family of 23 single site PpcA mutants. In this review, we summarize the functional characterization of the native and mutant proteins. Mutants that retain the mechanistic features of PpcA and adopt preferential e^−^/H^+^ transfer pathways at lower reduction potential values compared to the wild-type protein were selected for *in vivo* studies as the best candidates to increase the electron transfer rate of *G. sulfurreducens*. For the first time *G. sulfurreducens* strains have been manipulated by the introduction of mutant forms of essential proteins with the aim to develop and improve bioelectrochemical technologies.

## Introduction

Biological processes have the potential to promote sustainable energy strategies and to deal with environmental contamination. The hallmark physiological characteristic of the *Geobacteraceae* family of bacteria is their ability to oxidize organic compounds completely to carbon dioxide with the concomitant reduction of various extracellular electron acceptors. These include the reduction of Fe(III) and Mn(IV) oxides, as well as the reduction of soluble U(VI) to insoluble U(IV). The latter process can be utilized for immobilization of uranium to prevent contamination of ground-water (Lovley et al., 1987, 1991; Lovley and Phillips, 1988). *Geobacter* species are also being explored to generate electricity from waste organic matter using electrodes as electron acceptors in microbial fuel cells (Nevin et al., 2008; Yi et al., 2009). The natural abundance of *Geobacter* species in distinct environments and their capability to perform extracellular electron transfer (EET) to reduce toxic/radioactive metals and to convert renewable biomass into electricity prompted their selection as target bacteria for practical biotechnological applications in the areas of bioremediation, bioenergy and biofuel production.

*Geobacter sulfurreducens* has 111 genes for *c*-type cytochromes, most of which contain multiple hemes (Methé et al., 2003). Gene knockout and proteomic studies identified several *c*-type multiheme cytochromes that participate in EET pathways. These include the inner-membrane heptaheme cytochrome ImcH, the five periplasmic triheme cytochromes of the PpcA family (PpcA-E) and several outer membrane cytochromes (Leang et al., 2003; Lloyd et al., 2003; Methé et al., 2003; Mehta et al., 2005; Holmes et al., 2006; Shelobolina et al., 2007; Ding et al., 2008; Kim and Lovley, 2008; Kim et al., 2008; Nevin et al., 2009; Levar et al., 2014; Liu et al., 2014; Smith et al., 2014). One methodology to develop and improve the bioremediation and electricity production capabilities of *Geobacter* species is to perform rational engineering of the relevant proteins for EET.

*G. sulfurreducens* expresses various periplasmic cytochromes (Lloyd et al., 2003; Ding et al., 2006) that occupy a strategic position to function as a capacitor (Esteve-Núñez et al., 2008) and control electron flow toward outer membrane components. Therefore, PpcA family cytochromes are in the front line as potential targets to develop mutant strains rationally designed to increase the respiratory rate of *Geobacter*. In order to achieve this goal, it is first necessary to obtain detailed structural and functional data for the targeted electron transfer components. However, the presence of several heme groups in multiheme cytochromes makes the determination of their solution structures difficult (Morgado et al., 2010b). In addition, the co-existence of several microstates connecting the fully reduced and oxidized states complicates the characterization of the individual redox centers and, therefore, of the functional mechanisms of multiheme cytochromes.

In this report, we review the technological and methodological improvements that have contributed to the functional and structural characterization of *G. sulfurreducens* multiheme cytochromes, using the PpcA family as a model. The structures of all these cytochromes in the oxidized state were determined by protein crystallography (Pokkuluri et al., 2004b, 2010). The solution structure of PpcA was determined in the reduced state by nuclear magnetic resonance (NMR) (Morgado et al., 2012b). The functional mechanisms of PpcA family cytochromes showed that the highly abundant PpcA can couple e^−^/H^+^ transfer and might contribute to the generation of a proton electrochemical gradient across the cytoplasmic membrane within the physiological ranges of pH and redox potential for *G. sulfurreducens* (Morgado et al., 2010a, 2012a). The well-established structural and functional properties of PpcA were used to design a set of 23 PpcA mutants as a first step toward improving the EET capabilities of *G. sulfurreducens*. The results obtained from the characterization of these mutants are also described in this review. Finally, we report the successful engineering of *G. sulfurreducens* strains to express selected PpcA mutants, a procedure that establishes a foundation for future evaluation of these strains in EET.

## Methodological improvements to assist the solution structural characterization of multiheme cytochromes

Structural determination is a crucial step in understanding the functional mechanisms of proteins with multiple redox centers. NMR spectroscopy enables the determination of protein structures in similar conditions to their physiological environment, providing information about the protein's internal motions and interactions with its redox partners in solution. However, the numerous proton-containing groups of the hemes in cytochromes and the magnetic properties of the heme iron, particularly in the oxidized state, complicate the assignment of the NMR signals (Morgado et al., 2010b; Paixão et al., 2010). Advances in protein expression protocols have contributed to increase the expression yields for mature multiheme cytochromes (Londer et al., 2002, 2005, 2006; Pokkuluri et al., 2004a; Shi et al., 2005) and, concomitantly, to overcome the traditional difficulties associated with the determination of solution structures using natural abundance samples. This rendered the isotopic labeling of multiheme cytochromes much more cost-effective (Fernandes et al., 2008), facilitated the NMR signal assignment procedure and provided the foundations to identify redox partners and map their interacting regions (Dantas et al., 2014). A methodology that enables the isotopic labeling of multiheme cytochromes exclusively in their redox centers was recently reported (Fonseca et al., 2012b) and is expected to be a valuable tool in the assignment of heme signals in very large multiheme cytochromes for which the overlap of signals in the NMR spectra is severe.

**Figure 1:**
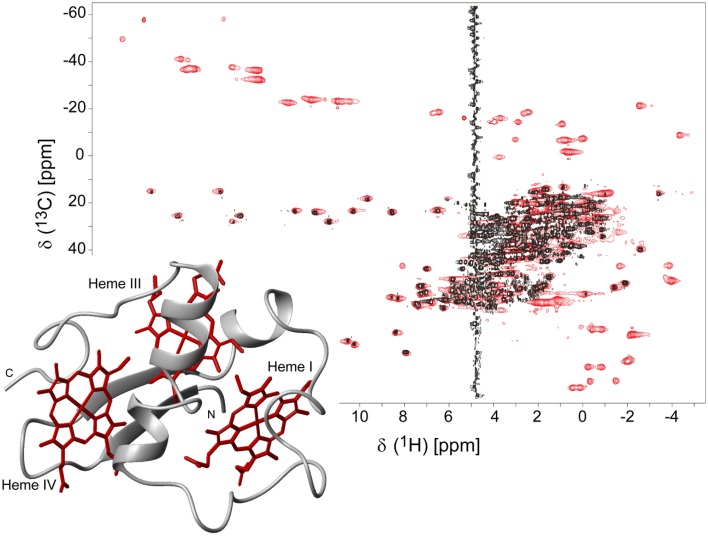
**2D ^1^H,^13^C HSQC NMR spectra of oxidized PpcA (pH 5.5 and 298 K)**. The spectra correspond to PpcA samples: labeled (^13^C/^15^N) exclusively in its polypeptide chain (black contours) and unlabeled sample (red contours). The red contours that are not overlapped with black contours correspond to the heme signals. The inset represents the lowest energy NMR solution structure of PpcA (PDB code 2LDO, Morgado et al., 2012b). The peptide chain and the hemes are colored gray and red, respectively. The hemes are numbered I, III and IV, a designation that derives from the superimposition of the hemes of PpcA and other cytochromes *c*_7_ with those of the structurally homologous tetraheme cytochromes *c*_3_.

The isotopic labeling protocol described by Fernandes et al. (2008) was used to produce ^15^N- and ^13^C,^15^N-labeled PpcA family cytochromes (Morgado et al., 2010b, 2011, 2012b; Dantas et al., 2015b) and allowed us to obtain in a cost-effective manner proteins labeled in their polypeptide chains with the correct folding and post-translational incorporation of heme groups. A strategy that simplifies the assignment of the NMR signals of multiheme cytochromes, developed by Morgado and co-workers (Morgado et al., 2010b), combines the analysis of ^1^H,^13^C HSQC (heteronuclear single-quantum coherence) NMR spectra obtained for an un-labeled sample and for a sample labeled exclusively in its polypeptide chain. A simple comparison of these spectra allows a straightforward discrimination between the heme and the polypeptide chain signals and is illustrated for PpcA in Figure 1.

**Figure 2:**
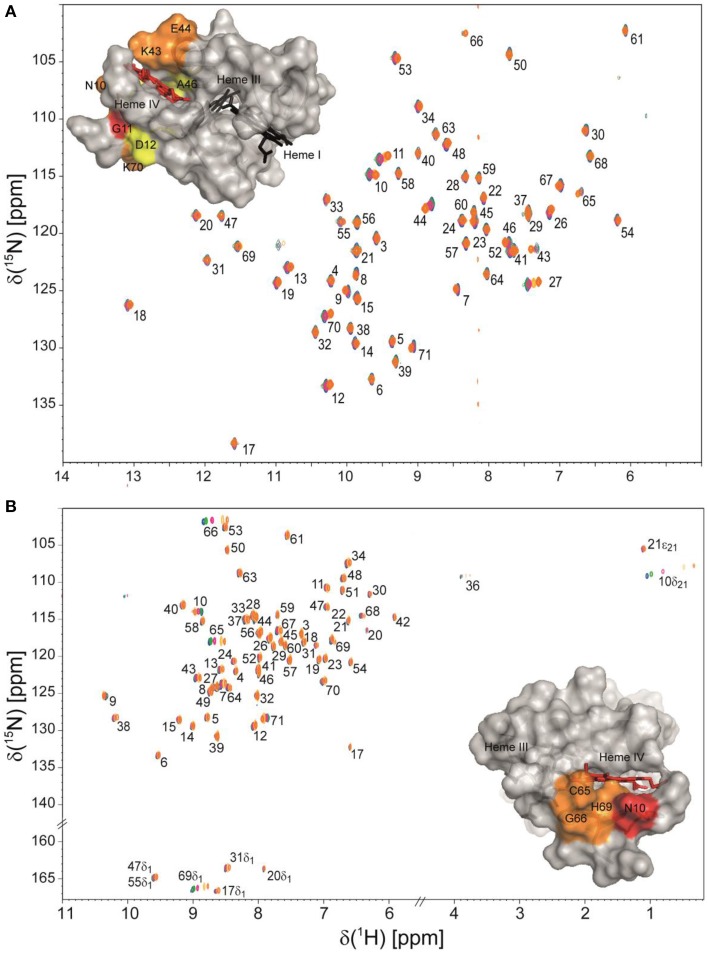
**Overlay of 2D ^1^H,^15^N HSQC NMR spectra of ^15^N-enriched oxidized (A) and reduced PpcA (B) in the presence of increasing amounts of the humic substance analog AQDS**. The assignments of NH signals are indicated. In the spectra the amount of AQDS increases from blue to orange contours. The insets show the surface map of significantly perturbed residues in PpcA (PDB code, 2LDO, Morgado et al., 2012b) upon binding of AQDS. The chemical shift perturbation increases from yellow (small) to red (large). Heme IV is shown in red and hemes I and III are shown in black. The molecular surfaces were generated in PyMOL (Wl, 2002).

Taking advantage of the methodologies described above, the NMR fingerprints, including heme proton signals and protein backbone and side-chain NH signals, were identified for the PpcA family cytochromes in both oxidized and reduced forms (Morgado et al., 2011; Dantas et al., 2014, 2015b). Chemical shift perturbation measurements on these fingerprints can quickly provide valuable information for investigation of EET respiratory mechanisms in *G. sulfurreducens*. Such investigations can involve protein-protein and protein-ligand interactions studies to survey and identify electron transfer between redox partners and establish foundations to engineer modified redox proteins for various applications. An example is provided for chemical shift perturbation studies carried out on PpcA at increasing concentrations of the humic substance analog anthraquinone-2,6-disulfonate (AQDS) (Figure 2). These studies showed that PpcA interacts with oxidized or reduced AQDS in the positively charged surface near heme IV (Dantas et al., 2014). When complemented with kinetic experiments, bidirectional electron transfer between PpcA and the humic substance analog was revealed for the first time. Such behavior might confer a selective advantage to *G. sulfurreducens*, which can modulate its energy metabolism according to the redox state of the humic substances available in the environment (Dantas et al., 2014). However, the precise mechanisms underlying the reduction of humic substances by *G. sulfurreducens* are still under debate. Lloyd et al. (2003) suggested that PpcA may transfer electrons directly to AQDS or humic materials that are able to traverse the outer membrane. On the other hand, Voordeckers et al. (2010) showed that the simultaneous deletion of genes coding for outer-membrane cytochromes OmcB, OmcS, OmcT, OmcE, and OmcZ yielded to a complete inhibition of AQDS reduction. Under the hypothesis that AQDS or humic substances are unable to access the periplasmic space of *G. sulfurreducens*, the interaction studies between AQDS and PpcA should be envisioned as working models (Dantas et al., 2014, 2015a). These models are even more relevant as no structural data are currently available for any of the outer membrane cytochromes mentioned above.

Isotopically labeled PpcA samples were also used to assist the determination of its solution structures in the oxidized and reduced states (Morgado et al., 2012b). Structural data are crucial to establish structure-function relationships, and therefore to optimize the rational design of electron transfer complexes that can improve the efficiency of microbial fuel cells and other *G. sulfurreducens* -based biotechnological applications (see below).

## Thermodynamic characterization of multiheme cytochromes

The reduction potential of a monoheme cytochrome can be obtained directly by the application of the Nernst equation, where *n* is the number of electrons involved in the reaction:

(1) $$\begin{array}{l} E=E^{0}+\frac{RT}{nF}ln\frac{\left[ox\right]}{\left[red\right]} \end{array}$$

**Figure 3:**
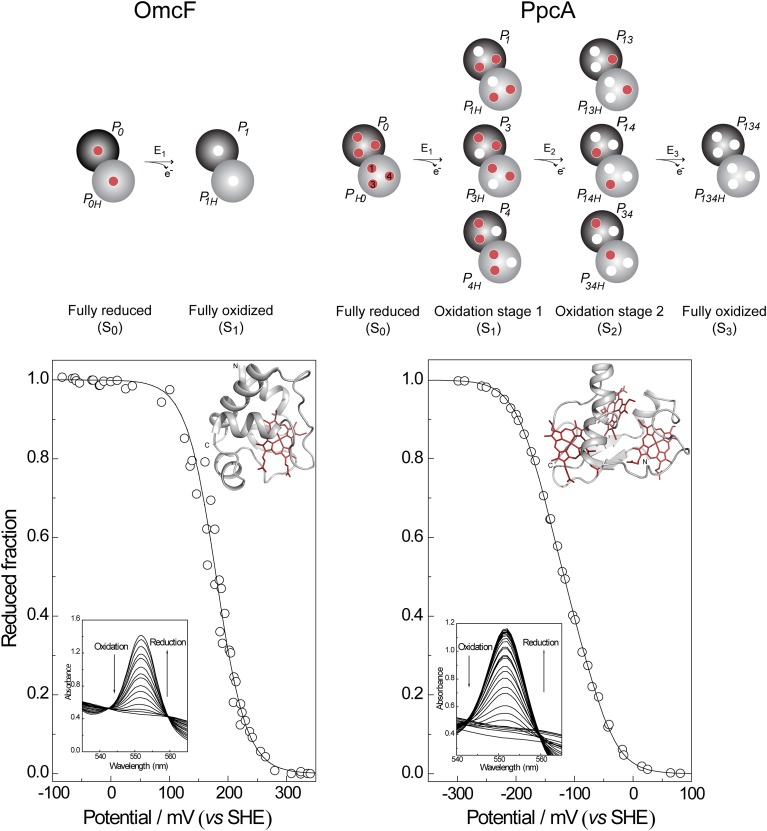
**Electronic distribution scheme for monoheme (OmcF) and triheme (PpcA) cytochromes showing the possible microstates in each situation**. The inner circles represent heme groups, which can either be reduced (red circles) or oxidized (white circles). The microstates are grouped according to the number of oxidized hemes in each oxidation stage, connected by consecutive one-electron redox steps. *P*_*0H*_ and *P*_*0*_ represent the reduced protonated and deprotonated microstates, respectively. *P*_*ijkH*_ and *P*_*ijk*_, indicate, respectively, the protonated and deprotonated microstates, where *i*, *j*, and *k* represent the heme(s) that are oxidized in that particular microstate. In the lower panels potentiometric redox titrations followed by visible spectroscopy of OmcF and PpcA (pH 7) are indicated. Solid lines indicate the result of the fits for the Nernst equation (OmcF) and for a model of three consecutive reversible redox steps between the different oxidation stages (PpcA) (Morgado et al., 2008; Pokkuluri et al., 2009). The structures of OmcF (PDB code, 3CU4, Pokkuluri et al., 2009) and PpcA (PDB code, 2LDO, Morgado et al., 2012b) are represented in the inset.

In this case, only the fully reduced and oxidized states co-exist in solution and the *e*_*app*_-value (i.e., the point at which the oxidized and reduced fractions are equal) corresponds to the reduction potential of the heme group. This is illustrated in Figure 3 for monoheme cytochrome OmcF from *G. sulfurreducens*. The fitting of Equation (1) to the data obtained from potentiometric redox titrations followed by visible spectroscopy for OmcF yielded an *e*_*app*_-value of +180 mV. On the other hand, redox titrations by potentiometric or electrochemical methods for multiheme cytochromes typically describe the whole-protein macroscopic redox behavior because the techniques cannot discriminate the reduction potentials of the individual redox centers. To illustrate this aspect, a potentiometric redox titration obtained for the triheme cytochrome PpcA is also represented in Figure 3. The experimental data can be fitted to a model that considers sequential midpoint reduction potentials for each center (Turner et al., 1996). However, this model is purely macroscopic and the reduction potentials obtained from the fitting (*E*_1_ = −171 mV; *E*_2_ = −119 mV; *E*_3_ = −60 mV), designated macroscopic reduction potentials, cannot be formally assigned to any specific heme in the protein. This is a consequence of the co-existence of several microstates in solution connecting the reduced and oxidized states. Figure 3 illustrates the microstates for a triheme cytochrome with one redox-Bohr center. In this case, the 16 microstates can be grouped according to the number of oxidized hemes in four macroscopic oxidation stages linked by successive one-electron reductions. For multiheme cytochromes containing higher numbers of hemes (N) and redox-Bohr centers (NB), the distribution can be easily scaled up. The total number of microstates and oxidation stages would be given by 2^N^(NB + 1) and N + 1, respectively. The microstates are interrelated by a set of Nernst equations and the mathematical formalism underlying the thermodynamic model that permits determination of the redox properties of the heme groups was first derived by Turner et al. (1996).

In addition to the pH of the solution (redox-Bohr interactions), the reduction potential of the hemes in multiheme cytochromes can be modulated by redox interactions with neighboring hemes, which can become quite significant when iron-iron distances are very short (Fonseca et al., 2012a). Therefore, for a triheme cytochrome the simplest model to describe the energy of the microstates over the full range of pH and solution potential, taking as reference the fully reduced and protonated protein, requires consideration of 10 parameters: the three energies of oxidation of the hemes (reduction potentials), the *pK*_*a*_ of the redox-Bohr center, and six two-center interaction energies (three heme–heme and three redox-Bohr). In order to achieve this it is necessary to monitor the oxidation profile of each heme at different pH values by NMR and complement this information with the data obtained from potentiometric redox titrations monitored by visible spectroscopy, as described by Turner et al. (1996).

## Thermodynamic characterization of PpcA family cytochromes from *g. sulfurreducens*

**Figure 4:**
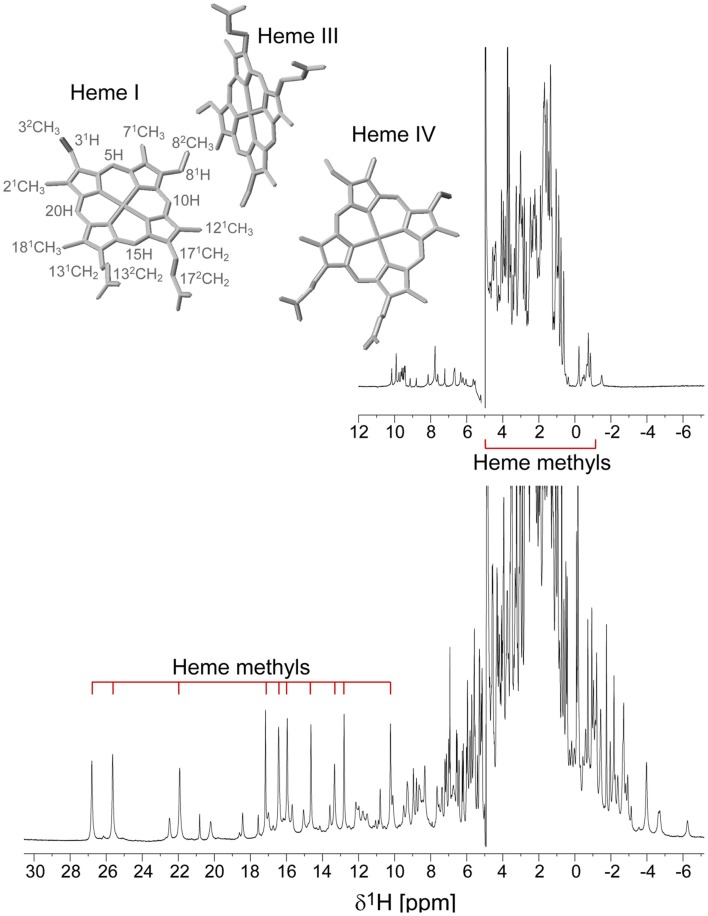
**1D ^1^H NMR spectra of the reduced (upper spectrum) and oxidized (lower spectrum) cytochrome PpcD (298 K)**. The typical regions of the heme methyl signals are indicated. In the oxidized spectrum the heme methyl signals are in the following order from left to right: 2^1^CH^I^_3_, 18^1^CH^I^_3_, 12^1^CH^I^_3_, 18^1^CH^IV^_3_, 12^1^CH^III^_3_, 7^1^CH^III^_3_, 7^1^CH^IV^_3_, 12^1^CH^IV^_3_, 2^1^CH^IV^_3_, 7^1^CH^III^_3_. The heme methyl 7^1^CH^I^_3_ and 18^1^CH^III^_3_, whose signals appear in crowded regions at chemical shifts of approximately 4 and −1 ppm, respectively, are not indicated. The inset indicates the heme core architecture of PpcD (PDB code 3H4N, Pokkuluri et al., 2010). The IUPAC nomenclature for tetrapyrroles is illustrated in heme I (Moss, 1988).

**Figure 5:**
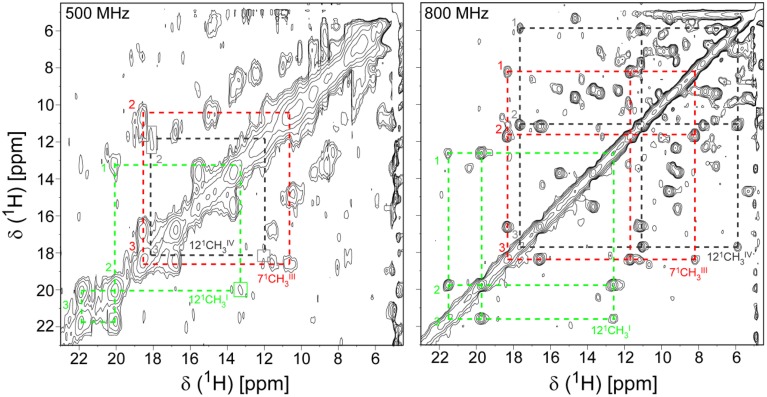
**Expansions of 2D EXSY NMR spectra of PpcA acquired at 500 MHz (500 mM ionic strength) and 800 MHz (250 mM ionic strength), at different levels of oxidation (288 K and pH 6)**. Cross-peaks resulting from intermolecular electron transfer across the oxidation stages 1–3 are indicated by dashed lines for the hemes 12^1^CH^I^_3_ (green), 7^1^CH^III^_3_ (red), and 12^1^CH^IV^_3_ (black). Signals connecting oxidation stage 1 are not visible at 500 MHz due the poor quality of this spectrum. Roman and Arabic numbers indicate the hemes and the oxidation stages, respectively. In order to prevent overcrowding of the figure, the 2D EXSY NMR spectra with cross-peaks to oxidation stage 0 are not shown.

The PpcA family cytochromes contain between 70 and 75 amino acids and three *c*-type hemes axially co-ordinated by two histidine residues (Pokkuluri et al., 2004b, 2010; Morgado et al., 2012b). The hemes are low spin in both diamagnetic (*S* = 0) reduced state and paramagnetic (*S* = 1/2) oxidized state (Morgado et al., 2010c; Dantas et al., 2011). These features are convenient in that they provide well-resolved ^1^H NMR spectra in both states. NMR explores the highly distinct features of the low-spin heme signals in the diamagnetic and paramagnetic forms. In the diamagnetic form, the chemical shifts of the heme substituents are dominated by the porphyrin ring-current effects and, therefore, appear in well-defined regions of the ^1^H NMR spectra (Figure 4) (Morgado et al., 2007, 2008, 2010a). In contrast, in the oxidized form the unpaired electron of each heme iron exerts significant paramagnetic shifts on the heme signals. Consequently, the same heme signals are differently affected by the paramagnetic centers, have different levels of broadening and are spread over the entire spectral width (Figure 4) (Morgado et al., 2010c; Dantas et al., 2011). As illustrated in Figure 4, the heme methyl signals are typically shifted to higher ppm values as the oxidation of the proteins progresses and, therefore, are ideal candidates to monitor the stepwise oxidation of the individual hemes throughout the different oxidation stages (see Figure 3). The heme methyl chemical shifts are proportional to the oxidized fraction of a particular heme, and therefore contain information about the redox properties of each heme (Morgado et al., 2010a). However, in order to probe the stepwise oxidation of the hemes, it is necessary to meet conditions of fast intramolecular electron exchange (between the different microstates within the same oxidation stage) and slow intermolecular electron exchange (between different oxidation stages) on the NMR timescale (Morgado et al., 2008). Slower intermolecular electron exchange can be favored by decreasing the temperature, decreasing the sample concentration or increasing the ionic strength of the solution. Earlier studies aiming to monitor the stepwise oxidation of the hemes in cytochrome PpcA were carried out at relatively high ionic strength (500 mM) and using a NMR spectrometer operating at 500 MHz (Pessanha et al., 2006). However, signal broadness was observed for the connectivities between the heme methyl signals in the different oxidation stages (Figure 5). The use of high-magnetic-field NMR spectrometers equipped with cryoprobes is an excellent example of how progress in the NMR technology has contributed to the detailed characterization of multiheme cytochromes (Morgado et al., 2010a). This equipment permitted the use of less concentrated samples (as low as 70μM) and low ionic strength solutions, favoring the slow intermolecular electron exchange regime between the different oxidation stages. Under these new experimental conditions, well-resolved 2D-exchange NMR spectroscopy (EXSY) spectra were obtained for PpcA family cytochromes (Figure 5). The stepwise oxidation of the hemes monitored by NMR experiments carried out in the conditions mentioned above, combined with data obtained from potentiometric redox titrations, allowed us to determine with higher precision the detailed redox properties of PpcA family cytochromes (Morgado et al., 2010a). The only exception was in the case of PpcC, which presented different conformations at intermediate stages of oxidation, leading to a splitting and an excessive broadening of the NMR signals, making it impossible to follow the oxidation profile of the individual hemes (Morgado et al., 2007).

## Functional mechanisms of PpcA family cytochromes

**Figure 6:**
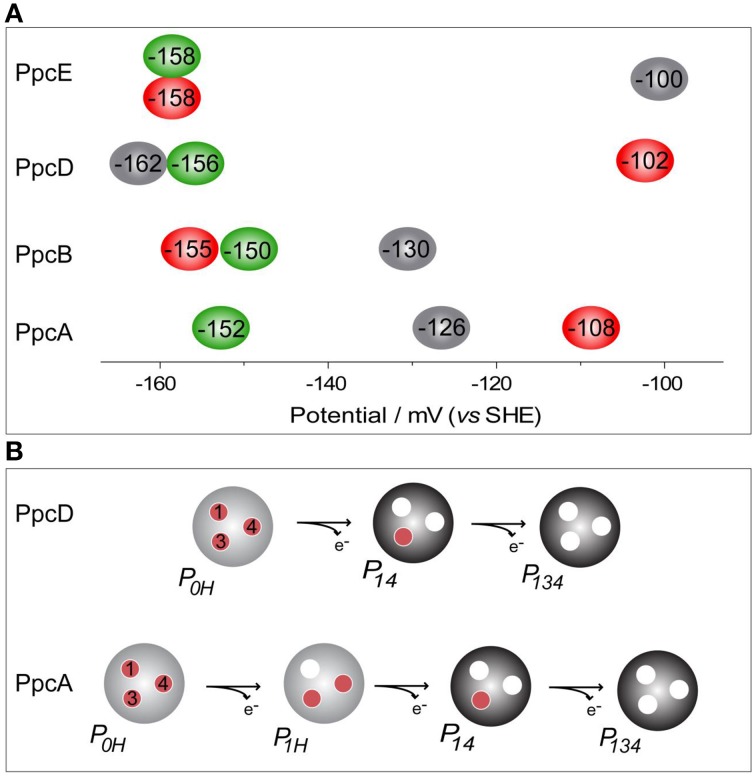
**Heme reduction potentials of PpcA, PpcB, PpcD and PpcE (A) and dominant microstates responsible for preferred e^−^/H^+^ transfer pathways observed for PpcA and PpcD (B)**. In **(A)** the hemes I, III, and IV are colored in green, red and black, respectively. Microstates in **(B)** are labeled as described in Figure 3.

The thermodynamic parameters obtained for the aforementioned cytochromes in the fully reduced and protonated state showed that the heme reduction potentials are negative, differ from each other, and cover different functional ranges (Table 1). These reduction potentials are strongly modulated by redox interactions amongst the three hemes (covering a range of 3–46 mV) and by redox-Bohr interactions between the hemes and a redox-Bohr center (2 to −58 mV), which was identified as heme IV propionate P_13_ (Morgado et al., 2008, 2012b). The positive values of the redox interactions indicate that the oxidation of a particular heme renders the oxidation of its neighbors more difficult, whereas the typically negative values observed for the redox-Bohr interactions show that the oxidation of the hemes facilitates the deprotonation of the redox-Bohr center and *vice versa*. Consequently, during the redox cycle of the protein the affinity of each redox center for electrons is tuned by the oxidation states of neighboring hemes and by the pH, such that their apparent midpoint reduction potentials (*e*_*app*_) are different compared to the values for the fully reduced and protonated protein. The *e*_*app*_-values for the heme groups of PpcA, PpcB, PpcD, and PpcE at physiological pH are indicated in Figure 6A. The contribution of each microstate in each oxidation stage (see Figure 3) can also be determined from the thermodynamic parameters listed in Table 1. The results clearly showed that PpcA and PpcD have dominant microstates during the redox cycle of the proteins (Figure 6B). In the case of PpcA, oxidation stages 0 and 1 are dominated by the forms *P_*0*_H* and *P_*1*_H*, respectively, in which the redox-Bohr center is kept protonated. Stage 2 is dominated by the oxidation of heme IV and deprotonation of the redox-Bohr center (*P*_*14*_), which remains deprotonated in stage 3 (*P*_*134*_). Therefore, a route is defined for the electrons within PpcA: *P_*0H*_ → P*_*1H*_ → *P*_*14*_ → *P*_*134*_. In the case of PpcD, a different profile for electron transfer is observed that favors a proton-coupled 2e^−^ transfer step between oxidation stages 0 and 2: *P*_*0H*_ → *P*_*14*_ → *P*_*134*_. In the case of PpcB and PpcE, several microstates are significantly populated in oxidation stages 1 and 2, and therefore no preferential pathway for electron transfer can be established. For further details on the fractional contribution of each microstate in each oxidation stage see Morgado et al. (2010a). The different functional mechanisms shown by the four periplasmic cytochromes indicate that they have evolved to perform different functions in the cell and illustrate how proteins with closely related structures can specifically fine-tune the properties of their redox centers.

**Table 1:** **Thermodynamic parameters for PpcA, PpcB, PpcD, and PpcE (Morgado et al., 2010a)**.

**Cytochrome**	**Energy (meV)**			
	**Heme I**	**Heme III**	**Heme IV**	**Redox-Bohr center**
**PpcA**				
Heme I	**−154 (5)**	27 (2)	16 (3)	−32 (4)
Heme III		**−138 (5)**	41 (3)	−31 (4)
Heme IV			**−125 (5)**	−58 (4)
Redox-Bohr center				**495 (8)**
**PpcB**				
Heme I	**−150 (3)**	17 (2)	8 (2)	−16 (4)
Heme III		**−166 (3)**	32 (2)	−9 (4)
Heme IV			**−125 (3)**	−38 (4)
Redox-Bohr center				**426 (8)**
**PpcD**				
Heme I	**−156 (6)**	46 (3)	3 (4)	−28 (6)
Heme III		**−139 (6)**	14 (4)	−23 (6)
Heme IV			**−149 (6)**	−53 (6)
Redox-Bohr center				**501 (8)**
**PpcE**				
Heme I	**−167 (4)**	27 (3)	5 (3)	−12 (4)
Heme III		**−175 (4)**	22 (3)	2 (4)
Heme IV			**−116 (5)**	−13 (4)
Redox-Bohr center				**445 (10)**

All energies are reported in meV, with standard errors given in parentheses. For each cytochrome, the fully reduced and protonated protein was taken as reference. Diagonal values (in bold) correspond to oxidation energies of the hemes and deprotonating energy of the redox-Bohr center. Off-diagonal values are the redox (heme–heme) and redox-Bohr (heme–proton) interaction energies.

## Rational design of PpcA mutant forms

**Figure 7:**
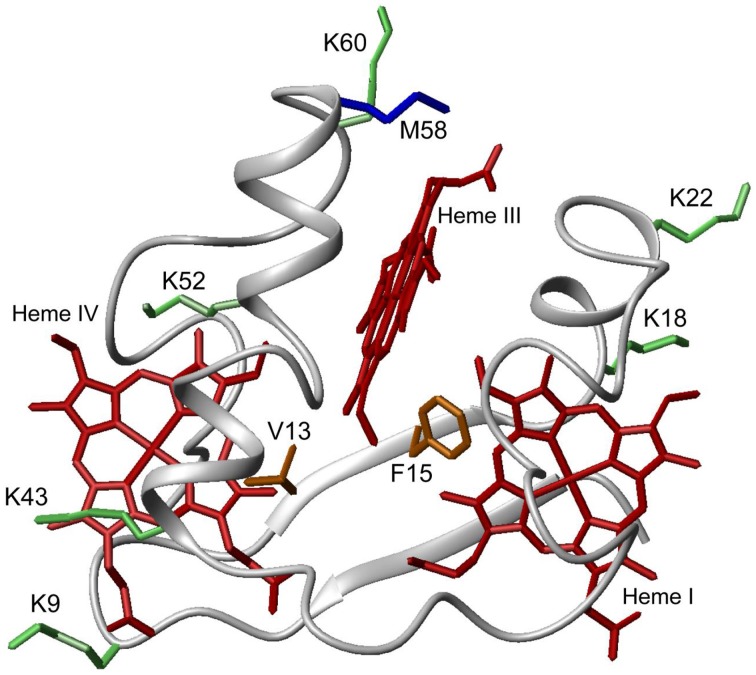
**Spatial location of residues mutated in PpcA solution structure (PDB code, 2LDO, Morgado et al., 2012b)**. The PpcA polypeptide chain (gray) is shown as Cα ribbon and heme groups (red). The side-chain of K9, K18, K22, K43, K52, and K60 (green); V13 and F15 (orange); M58 (blue) are represented as stick drawings.

PpcA is the most abundant cytochrome in *G. sulfurreducens* during growth on soluble and insoluble iron, and is most likely a reservoir of electrons destined for the outer surface (Ding et al., 2008). Therefore, it plays a crucial role by bridging electron transfer from cytoplasmic oxidation reactions to the reduction of extracellular terminal electron acceptors. The redox-Bohr effect in PpcA observed under physiologically relevant conditions, the magnitude of which is the highest amongst the PpcA family members (see Table 2), might implicate this protein in the e^−^/H^+^ coupling mechanisms that sustain cellular growth. In addition, structural data were obtained for this cytochrome in both oxidized and reduced forms (Pokkuluri et al., 2004b, 2010; Morgado et al., 2012b). For these reasons, PpcA was selected for rational design of mutant proteins, an essential step toward engineering variants of *G. sulfurreducens* with increased respiratory rates. A set of 23 PpcA-mutants was produced, following the protein expression and purification protocols previously described for the wild-type (Londer et al., 2002; Fernandes et al., 2008). The designed mutants covered sites in different regions of the protein and included: (i) conserved residues amongst PpcA family members located in the region of heme III (V13A, V13I, V13S, and V13T) and between hemes I and III (F15Y, F15W, and F15L); (ii) a residue M58 that was hypothesized to control the solvent accessibility of heme III (M58S; M58D; M58N, M58K) and (iii) lysine residues located near the hemes: K18 (near heme I), K22 (between hemes I and III), K60 (near heme III) and K9, K43 and K52 (near heme IV). In the last group of mutants, each lysine was substituted by glutamine and glutamic acid. The spatial location of each replaced residue is illustrated using the solution structure of PpcA (Figure 7).

**Table 2:** **Macroscopic *pK*_*a*_-values of the redox-Bohr center for PpcA, PpcB, PpcD, and PpcE in the reduced (*pK*_*red*_) and oxidized states (*pK*_*ox*_) (Morgado et al., 2010a)**.

	**PpcA**	**PpcB**	**PpcD**	**PpcE**
*pK*_*red*_	8.6	7.4	8.7	7.7
*pK*_*ox*_	6.5	6.3	6.9	7.4
*pK*_*a*_(*pK*_*red*_ − *pK*_*ox*_)	2.1	1.1	1.8	0.3

## Impact of the mutations on the global fold and heme core of the proteins

For each protein, the impact of the mutations on the global fold and heme core was probed by comparing the dispersion of both the heme proton and the polypeptide NH signals in 2D ^1^H-NOESY and ^1^H,^15^N-HSQC NMR spectra, respectively (Dantas et al., 2012; Morgado et al., 2013, 2014). For all the mutants, the protein global fold and heme core were conserved and changes in the chemical shifts were only observed for residues in the vicinity of the mutant site. It was possible to determine the heme oxidation profiles for 19 mutants (Table 3). On contrary, signal broadening was observed in the case of mutants F15W, F15Y, V13S, and V13T (Pessanha et al., 2004; Dantas et al., 2012), very likely due to the co-existence of more than one protein conformation in solution (Dantas et al., 2012), which impairs our ability to monitor the stepwise oxidation of the hemes. This suggests that the location of some residues in the 3D structure of the protein imposes severe constraints on the nature of the amino acids by which they can be replaced.

**Table 3:** **Variation of heme oxidation fractions of PpcA mutants *vs.* the native cytochrome (pH 6 and 8) at intermediate oxidation stages (*S*_1_ and *S*_2_) (Dantas et al., 2012; Morgado et al., 2013, 2014)**.

**Protein**	**Oxidation fraction relative to PpcA (%)**											
	**Heme I**				**Heme III**				**Heme IV**			
	**6**		**8**		**6**		**8**		**6**		**8**	
	***S_1_***	***S_2_***	***S_1_***	***S_2_***	***S_1_***	***S_2_***	***S_1_***	***S_2_***	***S_1_***	***S_2_***	***S_1_***	***S_2_***
V13A	−11	−9	−9	−9	7	3	7	7	4	5	9	4
V13I	3	2	1	2	−5	−5	−4	−6	2	3	4	4
F15L	−16	−4	−13	−8	16	19	16	22	−5	−16	−8	−15
M58S	1	0	0	1	−4	−4	−4	−4	2	3	4	2
M58D	−8	−3	−8	−5	8	9	9	11	−1	−6	−1	−6
M58N	13	6	7	9	−20	−29	−15	−25	8	22	11	16
M58K	11	4	6	7	−17	−24	−15	−22	7	19	9	14
K9Q	0	0	1	0	1	1	1	2	0	−1	−2	−2
K9E	−3	−2	−2	−1	−1	−5	−2	−4	4	7	1	6
K18Q	3	2	2	1	−1	2	0	2	−2	−4	−2	−3
K18E	6	3	6	2	−2	4	0	4	−4	−6	−6	−5
K22Q	−3	−1	−1	−1	3	2	3	3	0	−1	0	−1
K22E	−4	−1	−4	−2	5	6	6	8	−1	−5	−2	−5
K43Q	−5	−3	−6	−2	−1	−8	−2	−6	7	10	9	7
K43E	−20	−7	−22	−5	−9	−24	−11	−16	30	31	34	20
K52Q	−19	−8	−23	−6	−10	−24	−12	−16	29	31	36	20
K52E	−19	−7	−29	−10	−7	−23	−11	−13	29	28	38	21
K60Q	−8	−2	−7	−5	12	12	14	16	−3	−9	−7	−10
K60E	−13	−3	−14	−9	18	17	26	28	−4	−13	−11	−17

## Impact of the mutations on the relative heme oxidation profile

The redox-Bohr effect observed for PpcA showed that the heme oxidation fractions are modulated by the solution pH in the range 6–8 (Pessanha et al., 2006; Morgado et al., 2008, 2010a). Therefore, a survey of the heme oxidation profiles for each mutant was carried out at pH 6 and 8, as described for the wild-type cytochrome (Dantas et al., 2012; Morgado et al., 2013, 2014). This strategy allowed us to identify and select the mutants with considerable differences in their heme oxidation profiles compared to the wild-type for further detailed thermodynamic characterization. Comparison of the heme oxidation fractions, at both pH values, showed that the heme oxidation profiles were only slightly altered for V13A, V13I, M58S, K9, K18, and K22 mutants, whereas those for F15L, M58D/N/K, K43, K52, and K60 were significantly changed (Table 3). Therefore, the latter group of mutants was selected for detailed functional characterization (Dantas et al., 2012; Morgado et al., 2013, 2014). In addition, M58S was selected as an example from the former group of mutants and a detailed characterization was carried out on it to confirm that mutants showing no significant variation of their heme oxidation profiles displayed similar thermodynamic parameters and functional mechanisms compared to the wild-type.

## Impact of the mutations on the heme oxidation order at physiological pH

The detailed thermodynamic characterization of F15L, M58, K43, K52, and K60 mutants was carried out as previously described for the wild-type protein (Dantas et al., 2012; Morgado et al., 2013, 2014). The heme reduction potentials of these mutants are indicated in Table 4. As expected, the heme reduction potentials of the M58S mutant are similar to those of the wild-type cytochrome. However, considerable changes in the heme reduction potentials were observed for the other mutant proteins. In the case of the lysine mutants (K43, K52, and K60) the *e*_*app*_-values of the most affected hemes are smaller compared to the wild-type, as expected from the replacement of a positive charge in their vicinity. Similarly, the inclusion of a negative charge at position 58 is expected to stabilize the oxidized form of the nearest heme (heme III) by lowering its reduction potential. The opposite effect is expected by the introduction of a positive charge in the same position (M58K). All these effects can be rationalized on a purely electrostatic basis. On the other hand, the changes caused by the neutral leucine and asparagine side chains at positions 15 and 58, respectively, cannot be understood in purely electrostatic terms and were attributed to structural changes in the vicinity of heme III (Dantas et al., 2012, 2013; Morgado et al., 2013). With a few exceptions (K43Q, M58K/N), the changes observed in the heme reduction potentials also altered the oxidation order of the heme groups, compared to the wild-type (Table 4).

**Table 4:** **Comparison of the results obtained from the thermodynamic characterization of PpcA mutants at pH 7.5 (Dantas et al., 2012; Morgado et al., 2013, 2014)**.

**Protein**	***e*_*app*_ (mV)**			**Order of heme oxidation**	**Δ*e*_*app*_ (mV) (2^nd^/1^st^)**	**Δ*e*_*app*_ (mV) (3^rd^/2^nd^)**	**Electron transfer pathway**
	**Heme I**	**Heme III**	**Heme IV**				
PpcA	−152	−108	−126	I-IV-III	26	18	*P_0H_ → P_1H_ → P_14_ → P_134_*
K43Q	−162	−117	−150	I-IV-III	12	33	*P_0H_* (*P_1H_*) *P_14_ → P_134_*
K43E	−165	−117	−180	IV-I-III	15	48	*P_0H_ → P_14_ → P_134_*
K52Q	−157	−111	−175	IV-I-III	18	46	*P_0H_ → P_14_ → P_134_*
K52E	−156	−116	−177	IV-I-III	21	40	*P_0H_ → P_14_ → P_134_*
K60Q	−161	−143	−134	I-III-IV	18	9	No preferential pathway
K60E	−145	−146	−119	(III,I)-IV	1	26	No preferential pathway
M58S	−159	−110	−139	I-IV-III	20	29	*P_0H_ → P_1H_ → P_14_ → P_134_*
M58D	−160	−139	−140	I-(III,IV)	20	1	No preferential pathway
M58K	−159	−91	−146	I-IV-III	13	55	*P_0H_ → P_14_ → P_134_*
M58N	−163	−90	−152	I-IV-III	11	62	*P_0H_ → P_14_ → P_134_*
F15L	−155	−146	−125	I-III-IV	6	24	No preferential pathway
PpcB	−150	−155	−130	(III,I)-IV	5	20	No preferential pathway
PpcD	−156	−102	−162	IV-I-III	6	54	*P_0H_ → P_14_ → P_134_*
PpcE	−158	−158	−100	(III,I)-IV	0	58	No preferential pathway

For comparison the values previously obtained for PpcA, PpcB, PpcD, and PpcE (Morgado et al., 2010a) were also included. e_*app*_ (2^nd^/1^st^) is the difference between the e_*app*_-values of the second and the first heme to be oxidized. e_*app*_ (3^rd^/2^nd^) is the difference between the e_*app*_-values of the third and second heme to be oxidized.

## Impact of the mutated residues on the functional mechanism of PpcA

As described above, the oxidation profile of the redox centers is highly dependent on the nature of the side-chains at positions 15, 43, 52, 58, and 60. Thus, to evaluate the effect of each mutation upon the PpcA functional mechanism, the relative contributions of the 16 possible microstates were also determined (Dantas et al., 2012; Morgado et al., 2013, 2014). This information is summarized in Table 4 together with the data obtained for PpcA family cytochromes. As mentioned above, for PpcA a coherent e^−^/H^+^ transfer was established: *P_*0H*_ → P_*1H*_ → P_*14*_* → P*_*134*_*. In the mutant proteins different scenarios were observed. For K60, M58D and F15L mutants, lowering of the *e_*app*_* of heme III brings the midpoint reduction potential values of all the heme groups closer and favors the oxidation of heme III at earlier oxidation stages in a way that it is no longer the last heme to oxidize. Therefore, two microstates (*P_*1*_H* and *P_*3*_H*) dominated the first oxidation stage and, thus, no preferential pathway for electron transfer is observed for this set of mutants (Morgado et al., 2014). On the other hand, in the case of K43 and K52 mutants, the removal of a positively charged side-chain either at position 43 or at 52 contributes to stabilization of the oxidized form and lowers heme IV *e_*app*_*-values, so that it becomes the first one to oxidize, followed by heme I (Table 4). In these mutants, the oxidation stage 0 is also dominated by the protonated form *P_*0*_H*, as in PpcA, but the contributions of microstates in oxidation stage 1 are overcome by that of *P_*14*_* (microstate with hemes I and IV oxidized—see Figure 3). Thus, a different preferential route for electrons is established, favoring a proton-coupled 2e^−^ transfer step between oxidation stages 0 and 2: *P_*0H*_ → P_*14*_ → P_*134*_*, as observed for PpcD (Table 4). The same preferential electron transfer route is observed for M58K/N mutants. However, in this case, this is achieved by the concerted effect of increasing the reduction potential of heme III and decreasing that of heme IV (Table 4). Taking these observations together, it is clear that residues F15, K43, K52, M58, and K60 modulate the reduction potential of their closest hemes, which in turn controls the microscopic redox states that can be accessed during the redox cycle of the proteins.

**Figure 8:**
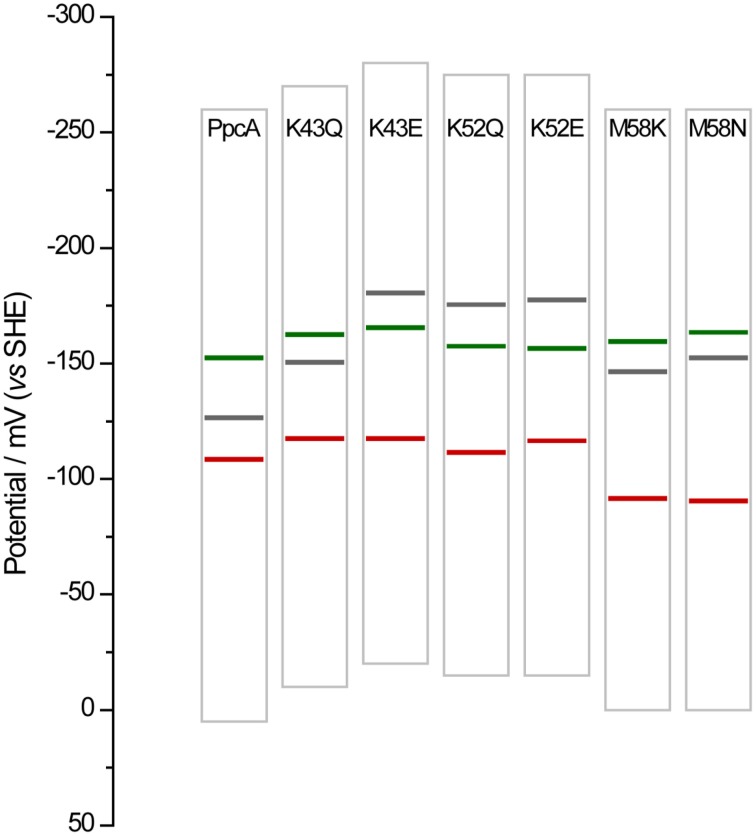
**Histogram comparison of the redox-active windows of wild-type PpcA and mutants showing increased differences between the *e_*app*_*-values of the third and second heme to oxidize Δ*e_*app*_ (3*^rd^/*2*^nd^)**. Horizontal lines represent the reduction potentials of the hemes I, III, and IV (see Table 4) colored in green, red and black, respectively. The potential windows were determined from potentiometric redox titration curves considering 1–99% of the range for protein reduction/oxidation.

Overall, the detailed study of this group of mutants suggests that the reduction potential of heme III, relative to the other two hemes, seems to be crucial in enabling these proteins to couple electron transfer with deprotonation of the redox-Bohr center. Indeed, preferential e^−^/H^+^ pathways are established only when heme III is the last one to oxidize (higher *e_*app*_*-value), which is reinforced by a higher separation between the *e_*app*_*-values of the second and third hemes to oxidize (see Table 4). However, the pathway varies with the separation between the *e_*app*_*-values of heme III and its predecessor in the order of oxidation (Table 4). In the case of PpcA such separation was 18 mV and the route for electron transfer was: *P_*0H*_ → P_*1H*_ → P_*14*_ → P_*134*_*. In the case of K43Q the same route was observed but with slightly higher separation between the *e_*app*_*-values (33 mV vs. 18 mV in the wild-type). Finally, in K43E, K52Q/E, and M58N/K the higher separation between the *e_*app*_*-values of heme III and its predecessor led to a significant contribution of the microstate *P_14_* so that a different preferred route for electrons was observed: *P_*0H*_ → P_*14*_ → P_*134*_*. It is important to note that this preferential route is independent of the order of oxidation of the first hemes (I-IV or IV-I). The impact of the changes in heme redox potentials, heme-heme interactions and redox-Bohr interactions on the behavior of the redox centers is summarized in Figure 8. The more negative values of the heme redox potentials compared to PpcA indicate that the functional working potential ranges in the mutants are shifted to lower redox potential ranges by preserving the concerted e^−^/H^+^ transfer and energy transduction features of the wild-type protein. This would thermodynamically favor the reduction of downstream redox partners and might have an impact in increasing the efficiency of the *G. sulfurreducens* respiratory chain. As shown above, this can be achieved by the rational design of mutants in the region of heme III or heme IV that lead to the modulation of their reduction potential values.

## Ppca mutant strains of *g. sulfurreducens*

**Figure 9:**
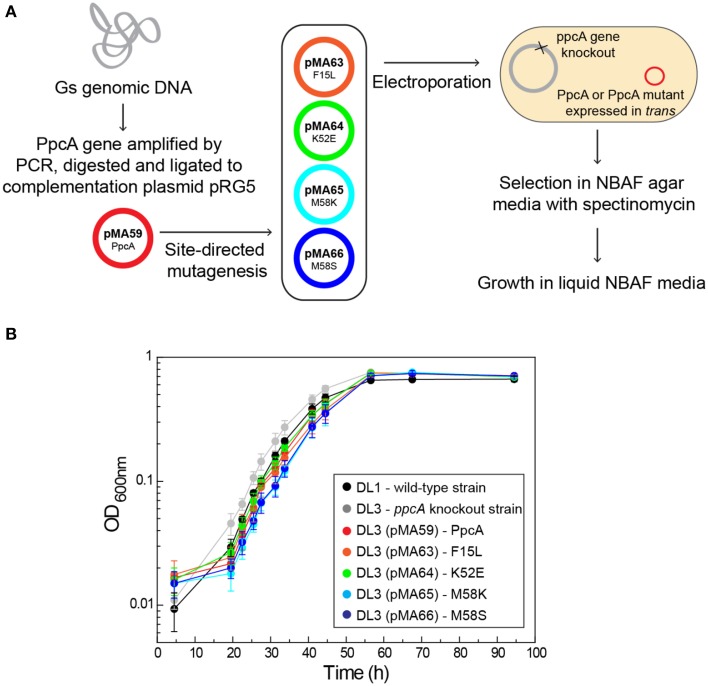
**Schematic representation of the preparation of *G. sulfurreducens* strains with mutated sequences of PpcA (A) and growth curves of *G. sulfurreducens* strains using acetate as electron donor and fumarate as electron acceptor (B)**. DL1, wild-type strain (black); DL3, *ppcA* knockout strain (gray); DL3 (pMA59), DL3 strain complemented with *ppcA* wild-type sequence (control, magenta); DL3 (pMA63), DL3 strain complemented with PpcAF15L sequence (orange); DL3 (pMA64), DL3 strain complemented with PpcAK52E sequence (green); DL3 (pMA65), DL3 strain complemented with PpcAM58K sequence (cyan); DL3 (pMA66), DL3 strain complemented with PpcAM58S sequence (blue).

The functional characterization of a large family of PpcA mutants provided a foundation to evaluate the effect that some of these mutants, when incorporated in *G. sulfurreducens*, would have on the electron transfer capabilities of this organism. Previous studies with a *G. sulfurreducens* strain with the *ppcA* gene knocked out showed that the growth with fumarate as an electron acceptor was not affected, but the growth rate significantly decreased when Fe(III) citrate was used as an electron acceptor (Lloyd et al., 2003). The ability to grow with Fe(III) was restored when PpcA was expressed in *trans* from a complementation plasmid. Taking into account the results obtained for the functional mechanisms of PpcA mutants, four mutants were selected for *in vivo* studies in *G. sulfurreducens*: (i) F15L that disrupts the preferential e^−^/H^+^ transfer pathway observed for PpcA; (ii) M58S, which conserves the functional mechanism observed for the wild-type; (iii) K52E and M58K, both of which favor a proton-coupled 2e^−^ transfer step but with different order of heme oxidation: IV-I-III and I-IV-III, respectively, at lower redox potential (see Table 4 and Figure 8). The *G. sulfurreducens* strains carrying the selected PpcA mutants were constructed using published protocols (Lloyd et al., 2003; Kim et al., 2005) and their viability was evaluated (Figure 9). The next step will be to study their growth in media with different electron acceptors, such as Fe(III) citrate and Fe(III) oxides. This work is currently underway.

## Outlook

In summary, the detailed analysis of PpcA mutants enabled us to learn the principles by which the individual redox properties of the hemes control the e^−^/H^+^ transfer pathways in multiheme cytochromes. It is expected that introduction of the mutations associated with preferential e^−^/H^+^ transfer pathways at lower reduction potential values in *G. sulfurreducens* cells will improve the bacterium's electron transfer rate, resulting in increased biomass yield. These features can be further explored to test the effect of *G. sulfurreducens* mutants on the efficiency of microbial fuel cells. In principle, this knowledge will enable us to design mutants of other cytochromes to achieve a specific desired functional pathway for any given biotechnological application.

### Conflict of interest statement

The authors declare that the research was conducted in the absence of any commercial or financial relationships that could be construed as a potential conflict of interest.

## Abbreviations

AQDS: anthraquinone-2,6-disulfonate
EET: extracellular electron transfer.
